# Reject Analysis in Digital Radiography and Computed Tomography: A Belgian Imaging Department Case Study

**DOI:** 10.5334/jbsr.3259

**Published:** 2023-12-22

**Authors:** Laura Haddad, Hanna Saleme, Nigel Howarth, Denis Tack

**Affiliations:** 1 Universitélibre de Bruxelles, Belgium; 2 Department of Radiology, Epicura La Madeleine, Rue Maria Thomée, 1, 7800 Ath, Belgium; 3 Department of Radiology, Hislanden –Clinique des Grangettes, 7 Chemin des Grangettes, 1224 Chênes-Bougeries, Switzerland

**Keywords:** Dose optimization, Effective dose, Dose-Length-Product, Dose-area-product, Computed Tomography, Digital, Radiography, Protocol optimization

## Abstract

**Objective:**

Reject analysis is usually performed in digital radiography (DR) for quality assurance. Data for computed tomography (CT) rejects remains sparse. The aim of this study is to help provide a straightforward benchmark for reject analysis of both DR and CT.

**Materials and methods:**

This retrospective observational study included 107,277 DR and 20,659 CT during 18 months in a tertiary care center. Rejected acquisitions were retrieved by Dose Archiving and Communication System (DACS). The DR and CT reject analysis included reject rates, reasons for rejection and supplementary radiation dose associated with these rejects.

**Results:**

8,904 rejected DR and 514 rejected CT were retrieved. The DR reject rate was 8.3% whereas the CT reject rate was 2.5%. The cumulative effective dose (ED) of DR rejects was 377.3 mSv while the cumulative ED of CT rejects was 1267.4 mSv. The major reason for rejects was positioning for both DR (61%) and CT (44%).

**Conclusion:**

This study helps constitute a simple reproducible method to analyze both DR and CT rejects simultaneously. Although CT rejects are less often monitored than DR rejects, the radiation dose associated with CT rejects is much higher, which emphasizes the need to systematically monitor both DR and CT rejects. Investigating the reasons and the most frequently rejected examinations gives an opportunity for improvement of imaging techniques in cooperation with technologists.

## Introduction

In medical imaging, radiation dose should be as low as reasonably achievable, also known as theALARA principle. Acquisitions must be “justified” and “optimized” according to guidelines [1]. Following this principle, any medical image associated with radiation and with no diagnostic value should be minimized as much as possible. Thus, it is recommended to regularly evaluate both compliance with procedures and reject rates of radiographic acquisitions [2]. This has been previously reported in many studies [3,4,5,6].

A rejected image refers to an acquisition that is considered unsatisfactory in terms of its quality by the radiographer at the time of capture. The radiographer takes another image fulfilling the technical requirements necessary for an accurate diagnosis [7,8]. Some studies refers to these images as repeats [8]. Reject analysis in digital radiography (DR) is an important part of quality assurance. While CT scans gives a larger ionizing dose, data available for CT reject analysis are limited [4].

The goal of this study is to help provide a straightforward benchmark for reject analysis of both DR and CT rejects in a single center. This analysis includes reject rates, reasons for rejection and supplementary radiation dose associated with these rejects.

## Materials and methods

This is an observational retrospective study, including 107,277 DR and 20,659 CT for 18 months, with complete anonymization of the data at the source. Informed consent was not required. Anonymous data regarding rejected CT and rejected DR acquisitions performed between April 1, 2021, and September 30, 2022, were extracted from the Picture Archiving and Communication System (PACS) in Telemis, Belgium and analyzed using the analysis software Intuitus Dose Archiving and Communication System (DACS). This software is specifically designed to make data about dose radiation and rejected acquisitions readily available for analysis. In recent literature, automated reject analysis algorithm is described to use data from Digital Imaging and Communications in Medicine (DICOM) to analyze deviations from standard acquisition to detect rejected images [8].

In our department of Diagnostic Radiology, at the occurrence of image rejection, the radiographer was asked to select the reason for the rejected image (Tables 2, 4). Technologists are trained to decide if the image processed is inappropriate for review by interpreting physician. In this manner, they automatically can select to reject an image. Regardless of the eligibility of the image for review, it is still saved to our Picture Archiving and Communication System (PACS). When the same kind of imaging is recorded twice the Intuitus DACS software highlights the flagged acquisitions with the rejection reasoning. A continuous quality control is performed on the Intuitus DACS software by a designated radiologist trained in radioprotection, which monitors rejected acquisitions related to each technologist and provide scheduled feedback to the team.

Usually, the technologist cooperates with the radiologist directly in case he has doubts if the image is sufficient to answer the clinical question, such as repeating a scanner with contrast injection to rule out a liver lesion. This is out of the scope of our study which focuses only technical errors leading to rejected acquisitions (Tables 2, 4). In the case of a wrongly imaged body part, it was considered a medical error and both images were sent for interpretation by the radiologist without classifying it as a rejected acquisition for technical issues.

A preliminary filtration was performed on Intuitus software to extract data flagged as rejects associated with technical issues, excluding repeated acquisitions for medical reasoning or during interventional procedures. These data were made ready to use after fine tuning by a team of developers from the Intuitus DACS application.

On Microsoft Excel spreadsheets, the data were then filtered according to body region.

DR and CT reject rates were calculated by dividing the number of rejects by that of the total number of examinations during that same period. First, overall DR and CT reject rate was obtained, then body region specific reject rates were obtained respectively.

To estimate the supplementary radiation dose associated with reject examinations, the effective dose (ED) of these acquisitions was calculated from primary radiation metric using conversion factors. The primary radiation metric for DR acquisitions was the dose-area-product (DAP, in Gy.cm^2^) [9]. For CT acquisitions, the primary radiation metric was the computed tomography dose index (CTDI, in mGy). The Dose-Length Product (DLP, in mGy.cm) which is equal to CTDI (mGy) times scan length (in cm), better represents the overall radiation output and subsequent potential biological effect attributable to the complete scan acquisition [1].

To obtain the radiation dose delivered by reject DR acquisitions, the cumulative dose area product (DAP) for each body region of rejected DR acquisitions was calculated. Conversion factors, also known as conversion coefficients (mSv/ Gy.cm^2^), were used to convert DAP (Gy.cm^2^) into effective dose (ED) (mSv). These conversion factors for DR imaging were selected from the International Commission on Radiological Protection (ICRP) Publication 103 (E-103). Wall et. Al expressed conversion factors E-103/DAP (mSv/ Gy.cm^2^) relating effective dose to DAP in a table including 24 types of radiographs [9]. For most x-ray examinations, two conversion factors are listed: a lateral (average of Left and Right Lateral) conversion factor and an anteroposterior (AP) or posteroanterior (PA) specific conversion factor. The DACS does not differentiate between AP and lateral images in the list of rejected images. To prevent underestimation of the dose, the highest conversion factor was used for each type of radiograph, to calculate the corresponding ED.

To obtain the radiation dose delivered by reject CT acquisitions, the cumulative DLP for each body region of rejected CT acquisitions was calculated. Conversion factors (mSv/mGy.cm) were used to convert DLP (mGy.cm) into ED (mSv) [10,11].

## Results

The overall DR reject rate was 8.3%. The most frequently rejected DR were knee DR (17.9%), hips DR (13%) and chest DR (10.5%) (Table 1). The cumulative DAP of rejected knee DR acquisitions was 145.3 Gy.cm^2^. A conversion factor of 0.0034 mSv/Gy.cm^2^ was used for rejected knee DR acquisitions to obtain the corresponding cumulated ED of 0.5 mSv [9]. Similar calculations were performed for rejected DR acquisitions of each body region (Table 1). The sum of the cumulative ED of the 8,904 rejected DR acquisitions, including all body regions, was 377.3 mSv (Table 1). The main reasons for DR rejects were positioning (61%), anatomy cut-off (21%) and clothing artefact (8%) (Table 2). The CT rejection rate was 2.5%. Head CT were the most frequent CT rejects with a head CT specific reject rate of 5.3% (Table 3). The cumulative ED of rejected head CT was of 232.8 mSv (Table 3). The sum of the cumulative ED of 514 rejected CT acquisitions, including all body regions, was 1,267.4 mSv. The main reasons for CT rejects were positioning (44%), anatomy cut-off (29%) and patient movement (8.9%) (Table 4).

**Table 1:** DR Reject Rate and corresponding radiation dose according to body region.


BODY REGION	REJECTED DR PER BODY REGION	DR ACQUIRED PER BODY REGION	DR REJECT RATE PER BODY REGION	CONVERSION FACTORS (MSV/GY.CM2)	REJECTED DR CUMULATIVE DAP (GY.CM2)	REJECTED DR CUMULATIVE ED (MSV)

Foot	401	6,974	5.7%	0.003	26.4	0.1

Knee	1,997	11,157	17.9%	0.003	145.3	0.5

Femur	55	710	7.7%	0.036	24.6	0.9

Hips	974	7,505	13.0%	0.130	379.8	49.4

Abdomen	80	1,648	4.9%	0.180	67.0	12.1

Pelvis	654	7,350	8.9%	0.140	500.2	70.0

Lumbar spine	659	20,766	3.2%	0.220	858.8	188.9

Thoracic spine	152	2,584	5.9%	0.240	74.3	17.8

Cervical spine	290	6,436	4.5%	0.190	25.0	4.8

Shoulder	811	15,303	5.3%	0.064	131.5	8.4

Chest	2,832	26,844	10.5%	0.160	152.6	24.4

Total	8,904	107,277	8.3%		2,385.3	377.3


Table 1 shows the number of rejected DR according to body region as well as the number of DR acquired per body region (including both accepted and rejected DR examinations). It also shows the conversion factors specific to each body region and the corresponding cumulative effective doses (ED).

**Table 2:** The identified reasons for DR image rejection.


DR REJECT REASON	N(%)

Positioning	5,431 (61%)

Anatomy cut-off	1,888 (21%)

Clothing artefact	712 (8%)

Under exposed	356 (4%)

Poor inspiration	338 (3.8%)

Over exposed	162 (2%)

Software failure	9 (0.11%)

Other failure	8 (0.09%)

Total	8,904


Table 2 shows the list of reasons for DR rejection and their percentage of occurrence.

**Table 3:** CT Reject Rate and corresponding radiation dose according to body region.


BODY REGION	REJECTED CT PER BODY REGION	CT ACQUIRED PER BODY REGION	CT REJECT RATE PER BODY REGION	CONVERSION FACTORS (MSV/MGY.CM)	REJECTED CT CUMULATIVE DLP (MGY.CM)	REJECTED CT CUMULATIVE ED (MSV)

HEAD	241	4,514	5.3%	0.0021	110,874.0	232.8

NECK	30	9,322	0.3%	0.0059	14,439.8	85.2

CHEST	22	2,041	1.1%	0.0140	3,389.0	47.4

ABDOMEN PELVIS	172	9,322	1.8%	0.0150	51,077.2	766.2

EXTREMITIES	49	1,184	4.1%	0.0110	9,050.3	135.8

Total	514	20,659	2.5%		188,830.3	1,267.4


Table 3 shows the number of rejected CT according to body region as well as the number of CT acquired per body region (including both accepted and rejected CT examinations). It also shows the conversion factors specific to each body region and the corresponding cumulative effective doses (ED).

**Table 4:** The identified reasons for CT image rejection.


CT REJECT REASON	N(%)

Positioning	225 (44%)

Anatomy cut-off	148 (29%)

Patient movement	46 (8,9%)

Inappropriate contrast phase	36 (7%)

Metal Artefact	29 (6%)

Poor Inspiration	20 (4%)

Software failure	5 (0.1%)

Other failure	5 (0.1%)

Total	514


Table 4 shows the list of reasons for DR rejection and their percentage of occurrence.

## Discussion

This was the first Belgium based study to perform both DR and CT reject analysis in the same radiological department. This study showed a DR reject rate of 8.3%, close to the target of 8% previously described in the literature, 10% being the threshold for corrective actions [2]. This study also showed a CT reject rate of 2.5%. In less recent studies, CT repeat rates concerned duplicate orders, for example in the case of transfer from another hospital, rather than repeated imaging for technical issues [12,13,14,15]. The study by Rose et al. performed a repeat analysis, similar to our reject analysis but focused on computed tomography pulmonary angiography (CTPA). Their results showed that CTPA repeat rates were 6.2 times that of all other CT examinations combined and that CTPA repeat rates were higher for large body patient protocol. They calculated the overall repeat rate among all protocols at each of the five sites which was less than 2%. Our results showed similar overall CT reject rate [8].

In another study, Rose et al. compared overall CT reject rate between two sites showing an average repeat rate of 1.2%, indicating better performance than our department. However, as stated in their study, low repeat rates can also be attributed to poor performance if the technologist does not recognize a non-satisfactory image and the need to repeat imaging [16].

In our study, we added the specific CT reject rate of each body region as well as the most common reasons for rejection to pinpoint the areas in need of improvement; this was also done for DR rejects. The most frequent type of rejected DR was knee DR (17.9%), hips DR (13%) and chest DR (10.5%). The study by Stephenson-Smith et al. showed that knee, hips, and chest DR acquisitions were also the most frequently rejected images. It would be beneficial to provide staff training on how to position patients for these projections. Conducting an educational in-service that focuses on relevant radiographic knee anatomy and positioning on X-ray is recommended [17].

The most frequently rejected CT examinations were the 241 head CT among the total 514 rejected CT, mostly related to patient movement, the third most common reasons for rejected CT (Table 4). The systematic use of fast acquisitions with higher pitch while minimizing image quality repercussion would be beneficial to the issue of patient movement. The shorter the duration of the acquisition, the lesser the chance of occurrence of patient movement. In their study, Rose et al. showed a significant difference at one site in axial overlap repeat rates compared with helical overlap repeat rates that could be related to a higher number of axial head scans at the emergency department [16]. The most common reason for rejected CT was positioning (44%) (Table 4). This issue could be improved with specific staff training as previously stated for rejected DR.

Performing a reject analysis raises awareness to the radiological team about the reject rate and the reasons for rejection. This analysis identifies areas that require optimization. As the most common rejection reasons for both DR and CT examinations were due to errors in positioning and anatomy cut off, this may suggest that particular attention should be drawn to this issue during training of the radiological team. In addition, simplifying the image taking process may help the technologist perform more efficiently concerning these issues. For instance, to perform a CT examination, the many steps include adjusting the pitch, kilovolt, and tube current, all of which could be automated. This would liberate time for the technologist to focus on adequate communication and positioning of the patient. The benefit of optimization to reduce scanning rejection has been previously reported [18].

Guidelines have also been reported to reduce rejections in DR. Some guidelines include training technologists to improve position errors as well as putting posters in dressing rooms to remind the removal of artifacts. Using illustrations can be a way to better convey these guidelines to patients and technologists [19]. Small consistent changes and reminders can be the key to a sustainable improvement in image quality with minimal radiation exposure.

In our department a designated radiologist trained in radioprotection oversees quality control using the Intuitus DACS software to monitor reject rates related to each technologist. This is carried under the scope of personal performance improvement goals to avoid anxiety of negative repercussions and hiding rejects [20]. Keeping the rejected acquisitions in the PACS is not necessary for calculating reject rates but it can be useful if one technologist is associated with high reject rate to review the rejection appropriateness concerning these rejected images. In parallel, exceptionally low reject rate associated with a technologist can also be a sign of poor performance and this is also carefully monitored [16].

To improve reject analysis, monitoring solutions could be modified on DICOM, PACS or DACS for simple access to reject rates [20]. The Intuitus DACS software used by our department accelerated and facilitated this reject analysis.

Another popular idea for optimization of CT scanning is using artificial intelligence (AI) techniques. Using a three-dimensional infrared camera and AI algorithms can minimize errors in centering and scan range during patient positioning which showed to be the main cause of rejected CT acquisitions in our study. AI deep learning (DL) based algorithms can also remove the noise and improve image quality [21]. AI can facilitate technologists work by simplifying the techniques to obtain high quality images, but there are more benefits of using AI. The most beneficial use of AI technology is that it give the technologists more time to focus on the human aspect of image processing. By spending more time on explaining and reassuring the patient, the patient will gain an improved experience in the imaging department as well as minimizing errors and higher radiation doses.

Our reject analysis also included the supplement dose delivered by these rejects. Despite CT rejects occurring less often than DR rejects, the cumulative radiation dose of 1,267.4 mSv delivered by CT rejects was around three times higher than that of DR rejects at 377.3 mSv. This highlights the importance to systematically perform CT reject analysis in parallel to DR reject analysis as it is the responsibility of the radiologist to follow the as low as reasonably achievable (ALARA) principle and minimize patient radiation dose [1].

We acknowledge several limitations in our study. Using data from a tertiary medical center with a diverse population resulted in very few pediatric patients being included as they are usually redirected to specialized centers in the area. There is also a risk for reporting bias since technologists manually select the reason for rejection. In addition, the list of reject DR images did not include specifically if the DR images were antero-posterior or lateral images. While underestimation of the dose was minimized by using the highest conversion factor, a suboptimal accuracy persists concerning the specific positioning, which is prone to rejection.

## Conclusion

The findings of this study demonstrated that reject analysis in both DR and CT is necessary in identifying areas for quality improvement. Technical deficiencies were identified using the analysis, which allows for recommendations and quality optimizations to be made. A potential direction for future research would be to conduct additional audits of the department after implementing the optimization and training strategies, which would reduce unnecessary radiation exposure. CT reject analysis is essential since the associated radiation dose is significant. The method used in this study is easily reproducible, providing a straightforward benchmark for future DR and CT reject analysis.
